# Zoonotic Hepatitis E Virus: An Ignored Risk for Public Health

**DOI:** 10.3389/fmicb.2017.02396

**Published:** 2017-12-04

**Authors:** Yuchen Nan, Chunyan Wu, Qin Zhao, En-Min Zhou

**Affiliations:** ^1^Department of Preventive Veterinary Medicine, College of Veterinary Medicine, Northwest A&F University, Xianyang, China; ^2^Scientific Observing and Experimental Station of Veterinary Pharmacology and Diagnostic Technology, Ministry of Agriculture, Xianyang, China

**Keywords:** hepatitis E virus, zoonosis, animal reservoirs, HEV pathogenesis, cross-species transmission, host tropism

## Abstract

Hepatitis E virus (HEV) is a quasi-enveloped, single-stranded positive-sense RNA virus. HEV belongs to the family *Hepeviridae*, a family comprised of highly diverse viruses originating from various species. Since confirmation of HEV’s zoonosis, HEV-induced hepatitis has been a public health concern both for developing and developed countries. Meanwhile, the demonstration of a broad host range for zoonotic HEV suggests the existence of a variety of transmission routes that could lead to human infection. Moreover, anti-HEV antibody serosurveillance worldwide demonstrates a higher than expected HEV prevalence rate that conflicts with the rarity and sporadic nature of reported acute hepatitis E cases. In recent years, chronic HEV infection, HEV-related acute hepatic failure, and extrahepatic manifestations caused by HEV infection have been frequently reported. These observations suggest a significant underestimation of the number and complexity of transmission routes previously predicted to cause HEV-related disease, with special emphasis on zoonotic HEV as a public health concern. Significant research has revealed details regarding the virology and infectivity of zoonotic HEV in both humans and animals. In this review, the discovery of HEV zoonosis, recent progress in our understanding of the zoonotic HEV host range, and classification of diverse HEV or HEV-like isolates from various hosts are reviewed in a historic context. Ultimately, this review focuses on current understanding of viral pathogenesis and cross-species transmission of zoonotic HEV. Moreover, host factors and viral determinants influencing HEV host tropism are discussed to provide new insights into HEV transmission and prevalence mechanisms.

## Introduction

Hepatitis E virus (HEV) is a quasi-enveloped, single-stranded positive-sense RNA virus belongs to the family *Hepeviridae* (Smith et al., 2014; Nagashima et al., 2017). *Hepeviridae* is a highly diverse family that contains several viral species including zoonotic, anthropotropic, and animal-restricted HEV or HEV-like virus isolates (Nan and Zhang, 2016). Although the origin of *Hepeviridae* is a mystery, analysis of key protein domains encoded by HEV open reading frames (ORFs) and their homologs from other viruses furnish strong evidence suggesting that *Hepeviridae* arose as a consequence of an ancient recombination event. Specifically, recombination occurring within the junction between non-structural and structural protein encoding regions among the *Alphatetraviridae* and *Astroviridae* has been cited (Kelly et al., 2016). Of nine of 21 regions evaluated as part of a global burden of disease (GBD) study, ∼3.4 million symptomatic cases of hepatitis E have recently been observed annually, resulting in 70,000 deaths and 3,000 stillbirths (Dalton et al., 2015). Originally, HEV infection was thought to be solely restricted to humans, causing a self-limiting hepatitis with mortality ranging from 0.5 to 3% overall, but mortality in pregnant women approaching 30% (Pillot et al., 1995; Jameel, 1999). However, the discovery of HEV in swine in 1997 suggests HEV has a wider host range and is actually zoonotic (Meng, 2013).

Currently, hepatitis E cases are frequently reported in developed countries and exhibit expanded host ranges (Doceul et al., 2016; Montesano et al., 2016; Park et al., 2016; Abravanel et al., 2017; Anheyer-Behmenburg et al., 2017). Thus, it appears that HEV has become one of the most successful zoonotic viral diseases (Dalton et al., 2015) and cross-species transmission of HEV from animal reservoirs to humans is the major route for HEV transmission in those countries (Pavio et al., 2015; Salines et al., 2017). Meanwhile, serosurveillance has demonstrated a high prevalence of HEV infection in the general population, which indicates the existence of an HEV endemic which has been underestimated for a long time (Sadik et al., 2016). Moreover, chronic HEV infection, HEV-related acute hepatic failure, and extrahepatic manifestations caused by HEV have been frequently reported in recent years (Dalton et al., 2016; Feng, 2016; Geng et al., 2016; Sadik et al., 2016). These observations suggest a complicated mechanism underlying HEV-related disease, especially for zoonotic HEV. Unfortunately, our understanding of HEV are extremely limited. Moreover, HEV is still less well known publicly as compared with other hepatic viruses such as hepatitis B and C viruses. In this review, recent progress made toward understanding zoonotic HEV host range, viral pathogenesis of zoonotic HEV, cross-species transmission of zoonotic HEV, and determinants influencing HEV host tropism are reviewed in detail and new insights are discussed.

## Classification of Diverse HEV Isolates

**FIGURE 1:**
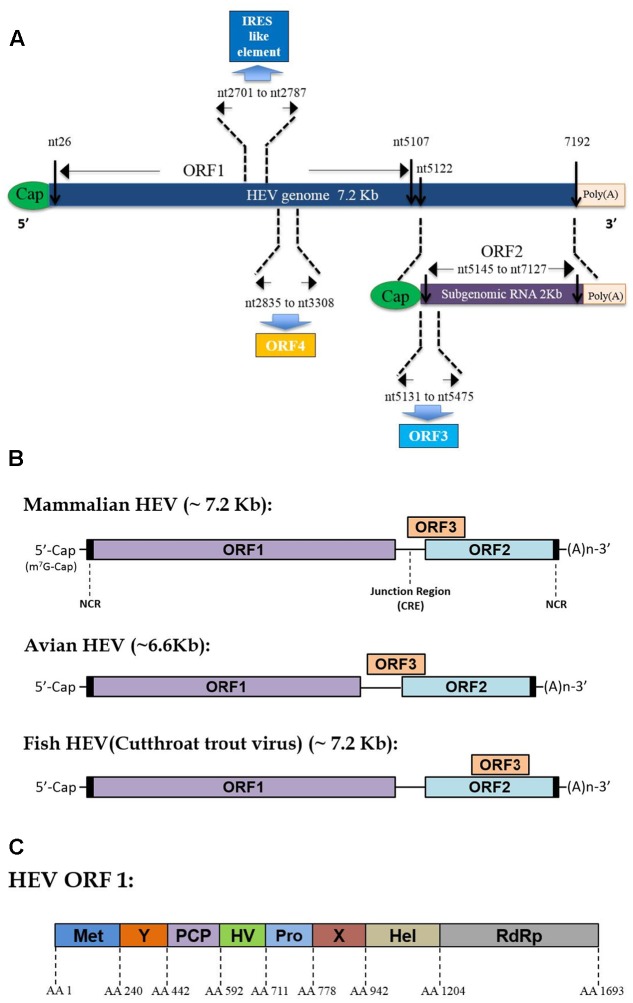
Hepatitis E virus (HEV) genome organization and function domain encoded by mammalian HEV ORF1. **(A)** Schematic illustration of HEV genome organization and subgenomic RNA. ORF1 (nt 26–5107) is labeled above the genomic RNA box. ORF2 (nt 5145–7127) and ORF3 (nt 5131–5475) are encoded by the same subgenomic RNA. The numbers above or below the RNA boxes indicate nucleotide numbers of the cDNA of HEV Sar55 (Genotype 1 *Orthohepevirus A* virus, GenBank accession # AF444002). **(B)** Genome location of ORF3 among different Hepevirus virus. **(C)** Schematic illustration of function domains encoded by mammalian HEV ORF1 polyprotein. Met, methyltransferase domain; Y, Y domain; PCP, papain-like cysteine protease; HV, hypervariable region; Pro, proline-rich domain; X, X-domain; Hel, helicase; RdRp, RNA-dependent RNA polymerase. The numbers above the box indicate amino acid residues encoded by of ORF1 of HEV Sar55 strain (Genotype 1 *Orthohepevirus A* virus).

Hepatitis E virus virions contain a 7.2 kb mRNA-like genome, which is capped and poly-adenylated (Ahmad et al., 2011). Currently, three well-recognized ORFs have been identified within the HEV genome for all genotypes (Tam et al., 1991; Tsarev et al., 1992), while the presence of an additional ORF4 has only been demonstrated in genotype 1 HEV so far (**Figure 1A**) (Nair et al., 2016). As an mRNA-like molecule, HEV-ORF1 is translated directly from its genome and encodes all non-structural proteins (mainly a replicase), which are essential for replication. Meanwhile, ORF2 and ORF3 can be only translated from the subgenomic RNA and partially overlap with each other (or completely overlap in some species of *Hepeviridae*) (**Figure 1B**) (Graff et al., 2006). ORF2 encodes the capsid protein (the major virion component), while ORF3 encodes a multifunctional protein (probably a class I viroporin), which is essential for virion release (Mori and Matsuura, 2011). Recently, synthesis of a novel ORF4 driven by a putative internal ribosome entry site (IRES)-like sequence was identified solely in genotype 1 HEV (Nair et al., 2016). Additionally, sequence analysis of other genetic elements has suggested that the HEV genome contains two *cis*-reactive elements (CRE) which are required for replication (Cao et al., 2010; Parvez, 2015). The second CRE is located in the intergenic region between ORF1 and ORF2 and forms a stem-loop structure which may serve as a promoter for subgenomic RNA synthesis (Cao et al., 2010).

After confirmation of HEV as the causative agent for hepatitis E, a prototype strain of HEV (SAR-55) originating from Pakistan was sequenced and served as the primary sequence for comparison to other isolates, such as the Burmese strain from India and several Chinese isolates (Aye et al., 1992; Tsarev et al., 1992; Bi et al., 1993). Sequences of these earlier HEV isolates shared the highest identity (greater than 90%) with each other and were classified as genotype 1 HEV (now known as HEV1 of *Orthohepevirus A* virus). Meanwhile, the HEV Mexican strain was originally viewed as the “New World” HEV strain due to its Central American source, while the Asian HEV isolate was viewed as “Old World” HEV (Reyes et al., 1991). Based on analysis of “Old World” and “New World” HEV sequences and excluding nucleotide sequences within the ORF1 hypervariable region, 84, 93, and 87% amino acid identity exists between the Mexican strain and the prototype Sar-55 strain for ORF1, ORF2, and ORF3, respectively (Huang et al., 1992). Subsequently, partial sequence analysis of newer HEV isolates from Africa has revealed that African HEV strains are distinct from the prototype Sar-55 strain, exhibiting greater similarity to the Mexican strain (Chatterjee et al., 1997). These novel isolates are classified as genotype 2 HEV (now HEV2 of *Orthohepevirus A* virus). However, only two complete sequences of genotype 2 HEV are currently available (Kaiser et al., 2017).

In 1997, a hallmark discovery affecting HEV research dramatically was the identification of swine HEV associated with human hepatitis E (Meng et al., 1997). Previously, phylogenetic analyses suggested that swine HEV is closely related to, but distinct from, previous known human HEV strains (the Asian prototype Sar-55 and the “New World” Mexican strain) (Meng et al., 1997). Subsequently, novel HEV isolates (strains US-1 and US-2) were recovered from two patients with acute hepatitis E in the United States and demonstrated close aa identity (>97%) with the 1997 US swine HEV isolate (Kwo et al., 1997; Schlauder et al., 1998). These isolates represent another HEV genotype (genotype 3 HEV, now designated HEV3 of *Orthohepevirus A* virus), which is zoonotic and causes cross-species infection (Kwo et al., 1997; Schlauder et al., 1998).

Next, in 1999 several new HEV isolates were obtained from Chinese HEV patients. These isolates were similar to one another but divergent from all other previously recognized HEV genotypes 1–3. Thus, they comprised a fourth HEV genotype based on phylogenetic analysis (Wang et al., 1999, 2000). Meanwhile, similar HEV isolates closely related to Chinese genotype 4 were soon reported in Japan and shown to cause indigenous acute hepatitis and consequently led to the establishment of a remarkably heterogeneous genotype 4 HEV group (Takahashi et al., 2002, 2003). Concurrently, a genotype 4 HEV isolate obtained from a sporadic acute hepatitis E patient demonstrated 99.0% sequence identity (nearly 100% identity) to the swine HEV swJ13-1 strain isolated from pigs (Nishizawa et al., 2003), supporting genotype 4 HEV as a zoonotic HEV genotype. Furthermore, although both HEV genotypes 3 and 4 are capable of infecting swine, the term “swine HEV” is confusing, since it actually neglects the existence of genotypic differences between HEV strains isolated from swine. Therefore, further clarification or genotype description is needed before using the term “swine HEV”.

In 2014, a newly proposed HEV classification system under the family *Hepeviridae* was published to provide a unified classification for diverse HEV isolates (Smith et al., 2014). In this proposal, two genera, *Orthohepevirus* (including all mammalian and avian HEV isolates) and *Piscihepevirus* (only trout HEV), exist under the family *Hepeviridae*. It is notable that all four major HEV genotypes (1–4) that infect humans are now assigned to the species *Orthohepevirus A* (Smith et al., 2014). Within *Orthohepevirus A*, two genotypes (5 and 6) represent novel HEV strains obtained from wild boar in Japan with unique viral nucleotide sequences that were previously designated as genotype 5 and genotype 6 HEVs (Takahashi et al., 2011, 2014; Smith et al., 2014). However, it should be noted that not all HEV isolates from wild boar belong to genotypes 5 and 6; indeed, most wild boar HEV isolates have been classified as genotypes 3 and 4 HEVs with the ability to infect humans (Sato et al., 2011). Moreover, other HEV strains isolated from camels in the Middle East with greater than 20% overall nucleotide difference from the other six HEV genotypes were categorized into genotype 7 within *Orthohepevirus A* (Smith et al., 2014; Woo et al., 2014). Notably, one patient from the Middle East who underwent liver transplantation had been infected by camel HEV, suggesting that camel HEV is zoonotic as well (Lee et al., 2016). Meanwhile, new hepatitis E virus isolates obtained from Chinese bactrian camels have recently been described and tentatively assigned to a new genotype 8, due to their genetic separation from genotype 7 camel HEV (Woo et al., 2016; Sridhar et al., 2017). However, this classification is not yet widely accepted. HEV-like isolates have constantly been discovered from additional hosts; based on the new classification system, rat and ferret HEV isolates have been categorized into *Orthohepevirus C*, while bat isolates have been classified into *Orthohepevirus D* (Lorenzo et al., 2007; Johne et al., 2010; Drexler et al., 2012; Raj et al., 2012; Krog et al., 2013; Lin et al., 2014; Smith et al., 2014; Woo et al., 2014). Notably, no zoonotic infections caused by *Orthohepevirus C or D* isolates have been reported to date in humans.

In addition to HEV genotypes that infect mammals described above, two unique HEV-like virus isolates from chicken and cutthroat trout (*Oncorhynchus clarkia*) are now assigned to species *Orthohepevirus B* and genus *Piscihepevirus* (Smith et al., 2014). Avian HEV-like viruses are also known as avian HEV (Zhao et al., 2010). Avian HEVs share less than 50% nucleotide identity to known mammalian HEVs (Haqshenas et al., 2001; Zhao et al., 2010). However, their viral capsid proteins contain both unique and common antigenic epitopes when compared with mammalian HEVs (Haqshenas et al., 2001, 2002; Huang et al., 2004; Guo et al., 2006; Zhou et al., 2008; Zhao et al., 2015). Consequently, the pathogenesis of avian HEV in chicken or hens is variable and may include hepatitis-splenomegaly syndrome (Payne et al., 1999; Haqshenas et al., 2001), decreased egg production (Zhao et al., 2017), or even subclinical infection (Gerber et al., 2015).

A distant cousin of HEV, cutthroat trout virus (CTV), was originally discovered in 1988 but has not been associated with any disease in these fish (Batts et al., 2011). In a retrospective study, CTV was identified as an HEV-like virus but shares even lower sequence identity with mammalian HEV than avian HEV; therefore CTV has been classified into a separate genus, *Piscihepevirus* (Batts et al., 2011; Smith et al., 2014). To date, comparison of CTV with HEV isolates from other hosts has not yet been conducted. However, to date no evidence has indicated that avian HEV or CTV is capable of infecting human or mammalian hosts.

## Function of HEV-Encoded Proteins

### Viral Replicase Encoded by HEV-ORF1 Protein

ORF1 is the largest ORF within the HEV genome (Tsarev et al., 1992). Originally, bioinformatics analysis of homologous domains encoded by ORF1 indicated the presence of at least eight putative domains that possess similarity to counterparts of other positive-sense RNA viruses (Koonin et al., 1992). These functional domains include RNA capping enzyme (including the previously known methyltransferase domain and Y domain), papain-like cysteine protease, hypervariable region (including previous known hypervariable region and proline-rich region), macro domain (also known as the X domain), RNA helicase, and RNA-dependent RNA polymerase domain (RdRp) (**Figure 1C**) (Ahola and Karlin, 2015; Nan and Zhang, 2016). Although the necessary protease cleavage sites to create functional ORF1-derived products are still unknown, available data indicate that these domains may function as independent units similar to replicases encoded by other positive-sense RNA viruses (Parvez, 2013, 2017; Paliwal et al., 2014; Nan and Zhang, 2016). Moreover, other studies suggest that cleavage of the HEV-ORF1 product may require participation of a papain-like cysteine protease domain encoded by the HEV-ORF1 itself (Parvez, 2013, 2017; Paliwal et al., 2014; Nan and Zhang, 2016).

One notable characteristic of HEV-ORF1 is its tolerance for nucleotide insertion and deletion within the hypervariable region. Initially, the hypervariable region was thought to serve as a hinge between the protease domain and the macro domain, since multiple proline residues within the hypervariable region form an unstable tertiary structure (Koonin et al., 1992; Tsai et al., 2001; Dosztanyi et al., 2006; Dunker et al., 2008). This putative function of the hypervariable region is also consistent with the variable length and sequence of the corresponding region in other HEV isolates (Pudupakam et al., 2009; Smith et al., 2013). Meanwhile, the hypervariable region appears to be an intrinsically disordered region (IDR) characterized by sustained gene segment insertion or deletion (Purdy, 2012; Purdy et al., 2012). Although deletion and mutation of the hypervariable region in HEV infectious clones suggests this domain is not required for viral replication and infectivity, the hypervariable region does play a role in HEV replication efficiency *in vitro* (Pudupakam et al., 2009, 2011). A remarkable HEV genotype 3 strain, Kernow-C1 p6, originally isolated from a chronically infected HEV patient (HIV positive), contains an insertion of a fragment (174 nt) of human ribosomal protein S17 obtained from its host (Shukla et al., 2011). This S17 insertion within HEV Kernoc-C1 p6 confers novel nuclear/nucleolar trafficking capabilities to HEV replicase, therefore enhancing viral replication *in vitro* (Kenney and Meng, 2015a,b). Moreover, although this region is interchangeable among HEV genotypes, genotype-specific differences of the hypervariable region imply that this region is involved in species tropism and host adaptation to HEV (Pudupakam et al., 2011).

### Viral Capsid Encoded by HEV-ORF2

Hepatitis E virus-ORF2 encodes the capsid protein, which is a major component of HEV virions. The full-length ORF2 product contains 660 aa residues with a predicted molecular mass of 72 kDa (Robinson et al., 1998). It also carries N-terminally linked glycans at three glycosylation sites (Asn137, Asn310, and Asn562) and a potential endoplasmic reticulum (ER)-directing signal peptide of about 15 aa within the N-terminus (Jameel et al., 1996). The mature HEV capsid protein requires proteolytic processing to remove the first 111 aa and the last 52 aa of the full length ORF2 and is able to form virus-like particles (VLP) when expressed in insect cells (Li et al., 1997, 2005). Genetic analysis of ORF2 suggests a greater than 85% similarity among HEV genotypes 1–4, with divergence mainly located within the first 111 aa of the N-terminus, a region which is not a known component of virions (Mori and Matsuura, 2011). Previous reports identified both conformational and linear neutralizing epitopes within HEV capsid protein, evidence that capsid protein is the major target of HEV neutralization (Gu et al., 2015; Tang et al., 2015). Therefore, a recombinant subunit vaccine based on truncated genotype 1 HEV capsid protein (HEV239) has been commercialized in China under the trade name Hecolin^®^ (Nan and Zhang, 2016). Meanwhile, avian HEV capsid protein sequences have been less thoroughly investigated due to their only 50% nucleotide identity with capsid sequences of the major mammalian HEV genotypes (Haqshenas et al., 2001; Zhao et al., 2010). However, avian HEV capsids do contain several linear epitopes which are shared with mammalian HEVs (Haqshenas et al., 2002; Guo et al., 2006; Zhou et al., 2008; Wang et al., 2015; Zhao et al., 2015), as well as epitopes only unique to avian HEV capsids (Wang et al., 2014). These results suggest the presence of antigenic variation between mammalian and avian HEV. Meanwhile, the concept that capsid proteins are the only structural proteins comprising HEV virions has been challenged by recent discovery of quasi-enveloped hepatitis E virus particles containing HEV-ORF3 product and a lipid bilayer (Nagashima et al., 2017).

Besides quasi-enveloped virions, review of historical data regarding HEV vaccine development based on VLPs raises additional questions about ORF2 precursor processing and VLP formation. Earlier studies demonstrated that expression of the ORF2 fragment (aa 112–608, genotype 1 HEV Burmese strain, 50 kDa) forms VLPs (Li et al., 1997, 2005). However, in one VLPs study of the genotype 1 HEV Sar-55 strain, expression of ORF2 in insect cells resulted in four ORF2-related protein products with sizes of 72 (full length), 63, 56, and 53 kDa (Robinson et al., 1998). Unlike the Burmese strain, the 53 kDa fragment (aa 112-578 of ORF2, the smallest product) of Sar55-ORF2 was able to form a VLP, while the 56 kDa ORF2 product (aa 112–607 of Sar55-ORF2) did not (Robinson et al., 1998). It is still unclear why the 53 kDa ORF2 fragment of Sar-55 was generated and why the 56 kDa Sar55-ORF2 fragment with similar aa sequence to Burmese strain 50 kDa fragment could not form VLPs. However, the 53 kDa VLP of Sar-55 appears to lack part of the putative neutralizing region of HEV-ORF2 located at aa 578–607 (Zhang et al., 2001). This result is consistent with partial protection of animals immunized with the Sar-55 53 kDa fragment VLP (Zhang et al., 2001), while immunization of the 56 kDa Sar55-ORF2 fragment (not the VLP) conferred full prevention of hepatitis upon intravenous challenge with homologous or heterogeneous genotype HEV strains (Purcell et al., 2003).

Conversely, in another study using aa 112–660 of genotype 1 HEV Burmese strain expressed in insect cells, neither the 53 kDa nor the 50 kDa ORF2 cleavage product was observed. Instead, two polypeptides with sizes of 73 and 62 kDa were produced (McAtee et al., 1996). Therefore, it appears that proteolytic processing and assembly of cleaved ORF2 products to form VLPs are much more complicated than currently understood. Moreover, although genotype 1 HEV is the most conserved HEV genotype, it still demonstrates variability in proteolytic processing and assembly of ORF2; therefore, further investigation is needed to determine if the same scenario also applies to ORF2 products of other genotypes, especially highly diverse genotypes 3 and 4.

### Multiple Function Protein Encoded by HEV-ORF3

It has been known for years that ORF3 partially overlaps with ORF2 in all mammalian HEV isolates and encodes a unique protein with undefined function (that contains protein motifs of known function). Two PSAP motifs have been identified for genotype 3 ORF3 product (aa 86–89 and aa 95–98), whereas genotypes 1, 2, and 4 lack the first motif (Nagashima et al., 2011b). Moreover, a phosphorylation site (Ser71) with unknown function was identified in genotype 1 HEV ORF3 that can be phosphorylated by a mitogen-activated protein (MAP) kinase (Zafrullah et al., 1997). Although ORF3 product (pORF3, hereby and thereafter) is not required for viral RNA replication *in vitro* (Emerson et al., 2006), it is indispensable for HEV infection *in vivo* and is essential for HEV virion release from infected cells (Graff et al., 2005; Huang et al., 2007; Yamada et al., 2009). In fact, most studies suggest that it is involved in viral release and formation of quasi-enveloped HEV virions from infected cells. Moreover, the second PSAP motif in pORF3 is needed for formation of membrane-associated HEV particles via pORF3 association with lipids (Nagashima et al., 2011a,b). Meanwhile, it is believed that pORF3 interacts with the tumor susceptibility gene 101 (TSG101), a component of the endosomal sorting complex required for creation of the transport (ESCRT) complex involved in budding of enveloped viruses. This interaction then leads to the biogenesis of quasi-enveloped HEV particles (Hurley, 2010; Feng et al., 2014; Nagashima et al., 2014; Yin et al., 2016). Notably, quasi-enveloped HEV particles are sensitive to detergent due to removal of the envelope (Yin et al., 2016), offering an explanation of why HEV particles obtained from fecal samples never exhibit envelope- and lipid-associated pORF3 (Nagashima et al., 2017).

Early in 2017, research of HEV-ORF3 identified that pORF3 shares key structural features with class I viroporins and functions as an ion channel that is essential for viral particle release during infection (Ding et al., 2017), which is consisted with putative role of pORF3 observed before (Yamada et al., 2009). Furthermore, a putative transmembrane region has been identified which may involve ER localization of pORF3 (Ding et al., 2017). This observation is interesting since viroporins could be a component of virions (as observed for the matrix-2 protein of influenza A virus) and may offer explanation of why antibodies against pORF3 product capture quasi-enveloped HEV particles (Takahashi et al., 2008; Nagashima et al., 2017). Moreover, pORF3 may be involved in neutralization of quasi-enveloped HEV particles, since immunization of bacterially expressed pORF3 could induce partial protection (Ma et al., 2009). Similar observations were confirmed by our lab in avian HEV (Syed et al., 2017). Together these data may provide novel concepts for use in future HEV vaccine development.

### Genotype 1 HEV-Specific ORF4

Recently, a novel ORF4 (nt 2835 to nt 3308 based on genotype 1 HEV) was identified within the HEV genome (Nair et al., 2016). Unlike HEV ORF1 to 3, translation of ORF4 is promoted by an atypical IRES-like element located in HEV-ORF1 along with a suboptimal Kozak sequence (Nair et al., 2016). Notably, it appears that HEV-ORF4 product is only conserved in genotype 1 HEV (Nair et al., 2016). While the exact function of pORF4 is still unclear, based on *in vitro* data ORF4 product expression stimulates ER stress which subsequently serves as a specific inducer of genotype-1 HEV replication (Nair et al., 2016). In addition, HEV-pORF4 is able to interact with multiple ORF1 domains to form a complex that enhances RdRp activity, which promotes viral replication as well (Nair et al., 2016). Furthermore, antibodies against HEV-pORF4 have been detected in HEV-infected patients (Nair et al., 2016). Nevertheless, additional investigation is needed to understand the function of the ORF4 product that is unique to genotype 1.

## The Zoonosis of Hepatitis E Virus

Hepatitis E was initially proposed to be a disease restricted to only developing countries (Aggarwal, 2011; Khuroo and Khuroo, 2016). However, this conclusion conflicted with the observations of sporadic hepatitis E cases in developed countries (Zaaijer et al., 1992; Coursaget et al., 1994; Tassopoulos et al., 1994; Zanetti and Dawson, 1994; Johansson et al., 1995). Although most sporadic hepatitis E cases in developed countries could be explained by transmission by travel from endemic regions or by contact with people traveling from an endemic region, HEV cases still appear and thus are not fully explained by the conventional fecal-oral transmission route. Therefore, HEV may retain a low endemicity in developed countries with unknown sources of infection (Tassopoulos et al., 1994). Meanwhile, after development of HEV diagnostic tools, serosurveillance data for anti-HEV antibodies (for detecting previous HEV infection) has revealed a drastically high proportion of the population (up to 28% in some areas) living in the United States and other developed countries where hepatitis E is not endemic (Lok et al., 1992; Quiroga et al., 1996; Mast et al., 1997; Thomas et al., 1997). Taken together, these observations imply that unknown sources of HEV infection or unrecognized non-pathogenic or lower pathogenic HEV strains are circulating in developed countries.

Although the exact causes of sporadic HEV cases in developed countries were unclear before the mid-1990s, several subsequent reports indicated that anti-HEV antibodies naturally existed in certain animal species such as primates (Arankalle et al., 1994), swine (Clayson et al., 1995), rodents (Turkestan rats, house mice, and wood mice), and sheep (Karetnyi Iu et al., 1993; Usmanov et al., 1994). Meanwhile, HEV RNA-positive samples were identified from stools of swine that were shedding virus (Clayson et al., 1995), which is consistent with an earlier report demonstrating that domestic pigs were susceptible to experimental infection by a human HEV strain of unknown genotype (Balayan et al., 1990). In other studies, because non-human primates are susceptible to human HEV infection, other animals were also tested for susceptibility to human HEV for establishment of animal models. However, only mixed data was obtained due to poor understanding of HEV genotypes and host tropism. For example, on the one hand lambs experimentally infected with a human HEV isolate (of unknown genotype, possibly genotype 1) contracted infection, with acute biochemical and histological evidence consistent with hepatitis (Usmanov et al., 1994). On the other hand, rats infected with human HEV (genotype was not mentioned) showed no clinical sign of hepatitis except evidence of HEV replication in the liver (Maneerat et al., 1996). Since viral nucleotide sequences in these animal studies were unavailable, no definitive evidence confirming the existence of a zoonotic HEV strain was reported before the mid-1990s. However, these earlier reports did imply possible HEV zoonoses had occurred.

Finally, in 1997 the hallmark of zoonotic HEV research was reported and described the identification and characterization of swine HEV isolates bearing a close relationship to human hepatitis E virus isolated from a swine farm in the United States (Meng et al., 1997). However, unlike human HEV, piglets infected with swine HEV were clinically normal except for microscopic evidence of hepatitis along with subclinical HEV viremia and seroconversion (Meng et al., 1997). Therefore, evidence for a direct link between swine HEV and human hepatitis E was emerging.

Coincidently, almost concurrent with the identification of swine HEV, two cases of human acute hepatitis E were reported in the United States. Notably, one patient had no epidemiological history of traveling from endemic regions (Kwo et al., 1997; Schlauder et al., 1998). Surprisingly, sequence of HEV (strain US-1 and US-2) recovered from these two patients demonstrated that both strains were quite distinct from previously known human HEV strains but shared highest similarity (greater than 97% aa identity of ORF1 and ORF2) to a swine HEV reference sequence (Meng et al., 1998b; Schlauder et al., 1998); this result finally confirmed an HEV zoonosis event. Moreover, both pigs and non-human primates could be experimentally infected by HEV US-2 human isolate or swine HEV isolate (Meng et al., 1998b). This result thus demonstrated that newly identified HEV isolates (swine HEV-related isolates) are zoonotic and capable of causing cross-species infection. Meanwhile, it is notable that experimental infection failed when human HEV strain Sar-55 (genotype 1) and Mexican strain (genotype 2) were used to inoculate pigs (Meng et al., 1998a). Collectively, these data confirm that at least some HEV strains are closely related to swine HEV can cross the species barrier to infect humans. On the contrary, pigs experimentally infected with either human HEV (Sar-55 and Mexican strain) or swine HEV were clinically normal. But seroconversion, fecal shedding of virus, and viremia were only observed in swine HEV-infected pigs, suggests a complete abolition of replication of human HEV (genotypes1 and 2) in pigs (Meng et al., 1998a). Taken together, these data indicate that zoonosis of HEV is genotype specific and does not apply to all HEV isolates despite the cross-reactivity of viral capsids between zoonotic HEV and human HEV.

After both genetic evidence and experimental evidence confirmed HEV zoonotic transmission, screening for HEV prevalence in various animal species, detection of new viral isolates, as well as identification of new hosts, were conducted worldwide. Consequently, the serum-positive prevalence of anti-HEV antibody was shown to be common among pig herds regardless of the HEV genotype circulating in human populations living within the same region (Chandler et al., 1999; Meng et al., 1999). Notably, HEV isolates from sporadic acute hepatitis E patients in Japan demonstrated 99.0% sequence identity (nearly 100%) to a swine HEV (swJ13-1) isolated from a pig living in the same region (Nishizawa et al., 2003). These isolates share highest homology with certain human HEV isolates from China, but were distinct from zoonotic swine HEV isolates from the United States. These results therefore suggest that zoonotic swine HEV strains are surprisingly heterogeneous and include various HEV genotypes (now known as genotypes 3 and 4 of species *Orthohepevirus A*) (Takahashi et al., 2002, 2003).

Meanwhile, rabbits may serve as a potential zoonotic reservoir for human hepatitis E as well (Burt et al., 2016). Based on sequence analyses of rabbit HEV strains isolated from the United States and China, rabbit HEV strains from both countries are closely related to genotype 3 HEV (Zhao et al., 2009; Cossaboom et al., 2011, 2012a). Moreover, a rabbit HEV strain identified from France matched a closely related human HEV isolate (from a French patient) and these isolates support possible zoonotic transmission of a genotype 3 rabbit HEV (Izopet et al., 2012). As further evidence, zoonosis of rabbit HEV was subsequently confirmed by experimental infection of cynomolgus macaques by rabbit HEV (Liu et al., 2013). Moreover, unlike swine HEV, experimental infection studies have suggested that rabbits are also susceptible to genotype 4 swine HEV (Ma et al., 2010; Han et al., 2014). However, no rabbit HEV isolates identified so far could be classified into genotype 4 HEV. On the contrary, although susceptible to rabbit HEV, pigs inoculated with rabbit HEV exhibited low levels of viremia and fecal virus shedding accompanied by active but weak HEV replication (Cossaboom et al., 2012b). These observations appear to be consistent with the few cases of rabbit HEV infection in humans, implying a potential species barrier separating rabbits from humans or other mammalian hosts to infection.

Deer and wild boar have been reported as potential zoonotic hosts for HEV as well (Tei et al., 2003; Takahashi et al., 2004). In one report from Japan, a full-genome genotype 3 HEV strain sequence obtained from wild boar demonstrated 99.7% sequence similarity with a previously characterized HEV strain originating from wild deer captured in the same region. The deer isolate was linked to virus from four hepatitis E patients who had eaten raw meat from a captured deer (Tei et al., 2003; Takahashi et al., 2004). Therefore, it appears that certain HEV strains of genotype 3 do not require genetic adaptation to cross the species barrier to infect new hosts. However, it is notable that no direct evidence suggesting a wild boar-to-human transmission pattern except the genetic identity of HEV recovered from patients and wild boar. Meanwhile, wild boar-specific HEV isolates (genotypes 5 and 6 of *Orthohepevirus A*) with unique nucleotide sequences have not yet been implicated in disease transmission to humans. Therefore, wild boar as a zoonotic host for HEV still needs further investigation.

More recently, while HEV and HEV-like virus have been consistently isolated from various animals, HEV surveillance data has suggested a greater host range for HEV than researchers originally predicted. Additional hosts for HEV include cattle, sheep, goats, cats, dogs, chickens, mongooses, bats, ferrets, rats, camels, and even trout (Liang et al., 2014; Smith et al., 2015b; Wu et al., 2015; Park et al., 2016; Yan et al., 2016). These new HEV isolates cannot be categorized within the old classification system of HEV and appear to form new genotypes, species, and even genera within the family *Hepeviridae* (Smith et al., 2014), suggesting *Hepeviridae* is a highly diverse viral family. Meanwhile, the zoonotic potential of these new HEV isolates is still unclear. To date, only one report has demonstrated a case of chronic HEV infection caused by genotype 7 camel HEV (Woo et al., 2014; Lee et al., 2016).

## Viral Pathogenesis of Zoonotic HEV

### Acute HEV Infection and HEV-Related Acute Liver Failure (ALF) in General Population

Hepatitis E virus infection in humans has been shown to cause acute hepatitis with a mortality rate from 0.5 to 3% in young adults (Jameel, 1999). Moreover, it appears that HEV is the most predominant cause of acute hepatitis in certain regions of developed countries (Kokki et al., 2016). The incubation period of HEV in human varies from 2 to 8 weeks (Purcell and Emerson, 2008) and initial clinical symptoms in acute hepatitis E patients appear to be non-specific and flu-like. After a short prodromal phase, hepatitis-specific symptoms such as vomiting, uncolored stools, darkened urine, and jaundice begin and may last from days to weeks. These symptoms are generally accompanied with evidence of liver damage, such as increased levels of liver transaminases, bilirubin, alkaline phosphatase, and blood γ-glutamyltransferase (Hoofnagle et al., 2012; Wedemeyer et al., 2012). Normally, acute hepatitis E is self-limiting, with full recovery in most cases. However, the most serious form, HEV-related fulminant liver failure, also known as acute liver failure (ALF), is reported in HEV-infected general populations (HEV-related ALF in pregnant women will discussed in a separated sub-section) and exhibits an extremely mortality rate approaching 50% if liver transplantation is unavailable.

Current understanding of HEV-related ALF remains elusive. A previous report suggested that genotype 4 HEV tends to cause more severe disease than others, suggesting that HEV genotype is the key determinant for HEV-related ALF (Jeblaoui et al., 2013). However, based on a literature search, HEV-related ALF caused by all conventional genotypes infecting humans has been reported (except for genotype 2) (Pujhari et al., 2010; Aherfi et al., 2014; Ramsay et al., 2015; Li and Chok, 2017; Wang and Geng, 2017). Although no direct evidence suggests a relationship between genotype 2 HEV infection and ALF, HEV-related ALF cases were identified in genotype 2 HEV endemic regions, which implies genotype 2 HEV could cause ALF as well (Maila et al., 2004). Considering the heterogeneous nature of genotype 3 and genotype 4 HEVs, these data suggest that host factors rather than viral factors such as viral genotype or mutation may contribute most to HEV-induced ALF (Smith and Simmonds, 2015). Meanwhile, a recent study suggested that HEV patients with pre-existing chronic liver disease tend to develop ALF (Wang and Geng, 2017), but compared to HEV-related ALF patients without chronic liver disease, no statistically significant difference was observed among two groups of patients. However, it should be noted that ALF caused by virus infection is neither solely a HEV-specific disease nor a hepatic virus (hepatitis A to E virus)-specific disease. Instead, ALF caused by either DNA or RNA viruses has been reported. These viruses include Epstein-Barr virus (So et al., 2007), varicella zoster virus (Vartian, 1999), coxsackie virus, and even dengue virus (Wallot et al., 2004; Osorio et al., 2008). Therefore, common host factors may be involved in virus-induced ALF caused by diverse viruses.

Conversely, interesting data has been reported for hepatitis B virus (HBV) supporting a Th2-dominated immune response that contributes to ALF induced by HBV. ALF associated with HBV infection is a dramatic clinical syndrome and exhibit a mortality rate approaching 80% (Farci et al., 2010; Wu et al., 2013). It had been observed very early that fulminant hepatitis B is characterized by an unusual antibody response to HBV antigens along with more rapid clearance than of than traditional acute hepatitis B (Farci et al., 2010). However, whether the unusual viral clearance and antibody response play a key role in HBV-associated ALF is unclear and has been debated for a long time. In a 2010 study conducted by Farci et al. (2010) analysis of tissue samples from two well-defined HBV ALF cases undergoing liver transplantation demonstrated that HBV-associated ALF is characterized by an overwhelming intrahepatic B cell response with massive accumulation of plasma cells secreting IgG and IgM along with complement deposition. Screening with phage display Fab libraries displaying both IgG and IgM from the liver tissue of two patients demonstrated that HBV core antigen was the primary target of these intrahepatic antibodies. Meanwhile, these antibodies displayed a restricted variable heavy chain (VH) repertoire and lacked somatic mutations (Farci et al., 2010). Sequencing data for antibody-coding genes suggested that these two unrelated individuals with HBV-related ALF possess an identical VH gene lacking somatic mutations within the variable domain (IGHV1-3) for generation of anti-HBV core IgG and IgM, suggesting that the HBV core antigen is targeted by a germline human VH gene in HBV-ALF patients (Farci et al., 2010). These data not only underlines the mechanism behind virally induced ALF but also suggests that genetic background might participate in ALF development during virus infection.

Although similar study is unavailable to examine if the same scenarios is applicable for HEV-related ALF, it has previously been demonstrated that patients with HEV-related ALF exhibit higher anti-HEV IgM and IgG titers than patients experiencing self-limiting HEV infections (Saravanabalaji et al., 2009). Consistent with this observation, another report demonstrated remarkably lower antiviral cellular immune responses in patients with fulminant hepatitis E than in patients with uncomplicated infections and controls (Srivastava et al., 2011). Taken together, these preliminary data appear to be strong evidence supporting pathogenesis of a Th2-dominated immune response in ALF induced by HEV, but this hypothesis awaits further investigation.

### HEV-Related ALF during Pregnancy

One remarkable characteristic of HEV infection is the high fatality rate resulting from HEV-related ALF in pregnant women during their third trimester of gestation (Bernuau et al., 2008; Shalimar and Acharya, 2013). Mechanisms underlying HEV-related ALF progression in pregnant women are still unclear, as no suitable animal model exists. For example, experimental infection of pregnant rhesus monkeys by intravenous administration of HEV does not result in increased severity of HEV-associated liver injury (Tsarev et al., 1995). One clue regarding HEV pathogenesis during pregnancy is that most cases are reported in India. This phenomenon might be partially explained by HEV genotype differences, since genotype 1 is thought to be the only HEV genotype found in Indian patients (Bernuau et al., 2008). Meanwhile, in another Indian report focusing on evaluation of HEV genotype associated with HEV-related ALF during pregnancy, only genotype 1 HEV was identified in that study (Kar et al., 2008). Moreover, although no data is available for evaluating genotype 4 HEV infection during pregnancy, two case reports from Europe demonstrated that genotype 3 HEV infection is not particularly lethal for pregnant women (Anty et al., 2012; Tabatabai et al., 2014). Therefore, focused research is needed to address if HEV-related ALF in pregnant women is especially restricted to genotype 1 HEV.

Regarding increased viral pathogenesis in pregnant patients, other clues have also surfaced. In one study, viral load in peripheral blood was found to be significantly higher in pregnant patients than in the non-pregnant control group (Kar et al., 2008; Bose et al., 2011). In another study, higher levels of TNF-α, IL-6, IFN-γ, and TGF-β1 were observed in pregnant vs. non-pregnant patients, suggesting that cytokine levels may be correlated with severe liver injury in HEV-infected pregnant women (Kumar et al., 2014). Moreover, a more recent study highlights the role of TLR3 and IFN-γ in HEV pathogenesis. Pregnant patients with high levels of TLR3 and a robust IFN-γ response exhibit acute viral hepatitis cases with limited disease progression and rapid recovery (Majumdar et al., 2015), whereas patients with lower expression of TLR3 and IFN-γ progress to ALF (Majumdar et al., 2015). This is consistent with another report demonstrating that down-regulated TLR3, TLR7 and downstream TLRs signaling molecules in pregnant ALF patients were correlated with defective innate immune responses, impaired macrophage function, and development of severe HEV-related ALF (Sehgal et al., 2015).

Also important, systematic changes due to sex hormones and immune response profiles during pregnancy, which tend to protect the fetus from rejection by the maternal immune system, may play a role in HEV-related ALF in pregnant women. Moreover, evidence supports a scenario involving a shift from a Th1-dominated immune response to a Th2-dominated immune response that protects the fetus from rejection (Romagnani, 1997). Meanwhile, as mentioned above, a Th2-dominated immune response contributes to ALF induced by HBV (Farci et al., 2010; Wu et al., 2013). Consistent with HBV-related ALF, existence of a Th2-biased immune response in pregnant women infected with HEV was previously demonstrated using measurement of cytokine production by peripheral blood mononuclear cells (PBMC). Furthermore, the results showed a reduction in Th1 cytokines and an increase in Th2 cytokines in HEV-infected pregnant women. However, the study’s implications for understanding the factors causing severe HEV infection in pregnant women are unclear, even though the results appear to agree with results for HBV-related ALF (Pal et al., 2005; Lhomme et al., 2016). Nevertheless, no further evaluation has yet been conducted to measure the intrahepatic immune response to determine if a pregnancy-induced Th2-dominated immune response participates in HEV-related ALF cases in pregnant women. Therefore, additional studies are urgently needed to answer this question and to compare the results to those from HBV-related AFL cases. In addition, the investigation of genotypic differences of HEV involved in HEV-related ALF during pregnancy is also needed.

### Chronic Hepatitis E Infection in Immunocompromised Patients

Initially, HEV was thought to only lead to acute infection as observed for hepatitis A virus, not chronic infection, as observed for HBV or HCV (Kamar et al., 2008). However, since the first identification of chronic HEV infection in patients receiving solid organ transplantation, chronic hepatitis E cases have been identified and are apparently associated with impaired immune function. Notably, there is one report shows that chronic HEV infection also occurred in an immunocompetent individual with systemic lupus erythematosus (SLE) (Grewal et al., 2014). However, since SLE with HEV is rare, data available so far is insufficient to support occurrence of chronic HEV infection in immunocompetent individuals (Kamar and Izopet, 2014), but is instead observed in immunocompromised persons such as organ transplant recipients, patients receiving cancer chemotherapy, and HIV-infected patients (Hoofnagle et al., 2012).

It appears that host immune status plays a key role in chronicity of hepatitis E infection since significantly lower CD2, CD3, and CD4 T cell levels were observed in patients who developed chronic disease (Kamar et al., 2008, 2011). Moreover, HEV-specific T-cell proliferation was decreased in transplant patients as well as in chronic hepatitis patients (Kamar et al., 2012; Suneetha et al., 2012; Brown et al., 2016). Meanwhile, the use of tacrolimus, a more potent immunosuppressant than cyclosporine A, has been associated with chronic HEV infection (Kamar et al., 2011). Taken together, these observations are consistent with results of a study showing that constant suppression of HEV-specific cell mediated immune responses by administration of immunosuppressants establishes chronic HEV infection in a swine model (Cao et al., 2017). In addition, a pilot study demonstrated that cynomolgus monkeys could be persistently infected by a genotype 3 hepatitis E virus isolate after long-term immunosuppressive drug administration, a result similar to that obtained in swine (Gardinali et al., 2017). Ultimately, these animal models will be useful tools for future development of therapy for chronic HEV infection.

It should be noted that chronic HEV infection appears to be restricted predominantly to genotype 3 and to a lesser degree genotype 4, whereas chronic HEV cases caused by genotype 1 and 2 have not yet been reported (Abravanel et al., 2014; Geng et al., 2014; Kamar et al., 2014). Moreover, it is interesting that chronic HEV infection in immunocompromised patients is linked to zoonosis and diversity of genotype 3 or even genotype 4 HEV. Such a bias may be partly related to the fact that genotype 3 HEV has a wider host range than other HEV genotypes (Smith et al., 2014). Meanwhile, one report demonstrated that genetic heterogeneity of HEV quasi-species within the ORF1 hypervariable region is much higher in chronic patients than patients who have cleared the virus (Lhomme et al., 2014b). Moreover, HEV isolates from chronic hepatitis E patients have been found to harbor viral-host recombinant variants with sequence variations within the hypervariable region (Shukla et al., 2011; Lhomme et al., 2014a). Fragments of human genes involved in these novel recombinant variants possess variable origins, which include ribosomal genes S17 or S19 and inter-alpha-trypsin inhibitor (Shukla et al., 2011; Lhomme et al., 2014a). Consequently, it appears such recombinant variants possess a replicative advantage *in vitro* (Nguyen et al., 2012). Therefore, chronic infection by HEV may confer greater diversity to the virus due to increased heterogeneity in the host. However, whether this increased heterogeneity is related to expansion of host tropism and HEV zoonosis is an interesting direction for future study.

### Pathogenesis of Zoonotic Hepatitis E Virus in Animals

In contrast to HEV infection in humans, no clinical evidence has demonstrated that animals infected with zoonotic HEV develop hepatitis. The reasons underlying this disparity are still a mystery. Moreover, for animal-specific HEV genotypes (excluding zoonotic HEV), the situation is similar as the only clinical hepatitis case reported for animals was in ferrets. When infected by ferret HEV, ferrets exhibit three hepatitis patterns: sub-clinical infection, acute hepatitis, and persistent infection (Li et al., 2016). However, another report demonstrated that ferret HEV isolates obtained from clinically healthy laboratory ferrets appeared to be non-pathogenic (Li et al., 2014). Therefore, it is still questionable if hepatitis observed in ferrets after inoculation with ferret HEV is due to a strain-specific cause of hepatitis or is a general consequence of ferret HEV infection. Meanwhile, since no evidence suggests that ferret HEV is zoonotic, the value of a ferret HEV model for human HEV remains unconfirmed.

In order to understand chronic HEV infection of animals, studies using the first swine HEV isolate identified (genotype 3) may provide clues. Although viremia and seroconversion were observed in experimentally infected piglets, piglets appeared clinically normal until microscopic examination for histological liver changes was performed (Meng et al., 1997). Moreover, zoonotic HEV isolates designated strains US-1 and US-2, which share over 97% aa identity to genotype 3 swine HEV, were obtained from patients with acute hepatitis (Kwo et al., 1997; Schlauder et al., 1998). Meanwhile, specific pathogen free (SPF) pigs inoculated with the US-2 strain were clinically normal without significant elevation of any liver enzymes tested, but did exhibit fecal shedding of virus and seroconversion, similar to clinical presentation observed of pigs inoculated with genotype 3 swine HEV (Meng et al., 1998b). Therefore, it appears that zoonotic HEV isolates (at least for genotype 3) demonstrated variable pathogenesis in different hosts, including acute infection in humans but subclinical infection in swine (Meng et al., 1998b). Moreover, when compared with genotype 1 HEV-infected non-human primates, liver enzyme elevation levels in serum samples of genotype 3 swine HEV-infected non-human primates were lower (Meng et al., 1998b). These results imply that most zoonotic HEV strains may exhibit lower pathogenicity than genotype 1 HEV.

Notably, a recent study on swine HEV co-infection with porcine reproductive and respiratory syndrome virus (PRRSV) demonstrated an interesting result. PRRSV is a swine-specific virus with a macrophage tropism that allows the virus to dampen the host immune system by inhibiting both innate immunity and cell mediated immunity (Nan et al., 2017). In this study, PRRSV-HEV co-infection in swine significantly extended the period of HEV shedding, established chronic HEV infection, and dramatically increased the chance that pork meat and livers contained HEV at slaughter, posing a potential risk for human infection (Salines et al., 2015). This observation is also consistent with results showing chronic HEV infection in swine after suppression of HEV-specific cell mediated immune responses using immunosuppressive drugs (Cao et al., 2017).

In addition to swine, HEV isolation has frequently been reported from rabbits. Both HEV genotypes 3 and 4 HEV infect rabbits (Cossaboom et al., 2012b; Liu et al., 2013, 2017). However, it appears that rabbit-HEV-infected rabbits are clinically normal because isolation of rabbit HEV from a SPF rabbit vendor is reported (Liu et al., 2017). Notably, another study has demonstrated that pregnant rabbits can be used to simulate the high mortality rate observed for human HEV infection during pregnancy (Xia et al., 2015). However, this result has not yet been confirmed by other researchers in rabbits or other animals. Nevertheless, rabbits may yet provide a novel model for understanding HEV pathogenesis in pregnancy.

## Cross-Species Transmission of Humans By Zoonotic HEV

To date, three zoonotic HEV genotypes have been confirmed to infect humans (Nan and Zhang, 2016). In addition to well-known HEV genotypes 3 and 4, genotype 7 of *Orthohepevirus A* (camel HEV) has also been recently demonstrated to infect humans (Lee et al., 2016). Moreover, analysis of the National Health and Nutrition Evaluation Survey (NHANES) 1988–1994 dataset found a relatively high seroprevalence (21%) of HEV infection in the United States general population (Ditah et al., 2014). Since most acute or chronic hepatitis E cases in developed countries have been sporadic and were caused by zoonotic HEV genotypes 3 and 4, it appears that cross-species transmission of zoonotic HEV from animal hosts to humans in developed countries depends on a variety of transmission routes other than the fecal-oral route. Nevertheless, the high stability of infectious HEV virions significantly increases the chance for cross-species transmission to humans regardless of route of transmission. Indeed, in one imported hepatitis E case reported in Japan, the HEV virus was believed to originate from an herbal product brought from China by the patient several months previously (Ishikawa et al., 1995). Furthermore, a recent study demonstrated that HEV virions obtained from cell culture maintained infectivity for up to 21 days at 37°C and for 28 days at room temperature (Johne et al., 2016). Therefore, it appears that HEV viral particles can remain highly stable in the environment (Purcell and Emerson, 2001).

After the discovery of swine HEV, the risk of zoonotic transmission has become a concern for public health. Most acute hepatitis E infections have been linked to consumption of contaminated pork or pig liver (Kamar et al., 2013; Pavio et al., 2014; Cossaboom et al., 2016). Moreover, the source of chronic hepatitis E infection in immunocompromised patients has often been unknown but is thought to be linked with pork consumption as well (Murali et al., 2015). Based on surveillance studies, approximately 2% of pig livers sold in Japan and 11% of pig livers sold in America are positive for HEV-RNA (Yazaki et al., 2003; Feagins et al., 2007). Moreover, deer have been demonstrated to be directly linked to zoonotic cases of hepatitis E in humans (Pavio et al., 2010). Meanwhile, since HAV could be taken up and concentrated by shellfish, recent studies have also supported shellfish consumption as a risk factor for HEV transmission and concentrated HEV virions have been detected in shellfish which is similar to HAV (Crossan et al., 2012; Gao et al., 2015; Huang et al., 2016). Therefore, it is important to study shellfish as a source of human HEV infection because they are filter-feeders. Such studies should be especially emphasized in more contaminated coastal areas, where consumption of raw shellfish may be a contributing factor to HEV incidence (Grodzki et al., 2014).

Contaminated milk obtained from animals might be another way for cross-species transmission of zoonotic HEV (Huang et al., 2016). Recently it has been discovered that camel HEV infection of an organ transplant recipient who regularly consumed camel meat and milk developed hepatitis, suggesting that HEV might have existed in the milk product (Lee et al., 2016). Although most milk for human consumption comes from cattle and goats, little research has been conducted to examine if HEV from cattle or goats is able to infect humans by this route. However, since both genotypes 3 and 4 HEV have been isolated from goats (Di Martino et al., 2016; Long et al., 2017), HEV transmission from goats to human is highly possible and goats should be considered as a potential animal reservoir for zoonotic HEV.

Besides foodborne transmission, direct contact with animal body fluids may be another transmission route for zoonotic HEV infection. It was reported earlier that HEV was detected in nasal and rectal swab materials in experimentally infected swine (Meng et al., 1998a). Meanwhile, during the experimental infection of rabbits with swine genotype 4 HEV, HEV-RNA was shed in the saliva of some rabbits as well (Wu et al., 2017). Moreover, a recent report documented that HEV-RNA was detected in the urine of experimentally infected pigs (Bouwknegt et al., 2009). Additionally, other data suggested that HEV could be transmitted to people by direct contact with infected animals (Andraud et al., 2013), which is consistent with observations of horizontal transmission from HEV infected wild boars that were in contact with pigs (Schlosser et al., 2014). Notably, these observations together offer a possible explanation for the rising rate of anti-HEV seroprevalence in swine workers and pork butchers (Traore et al., 2015; Lange et al., 2017; Teixeira et al., 2017).

In the fecal-oral transmission route, HEV are shed in the feces of infected individuals as stable, non-enveloped virions (Feng et al., 2014; Nan and Zhang, 2016). However, recent discoveries of quasi-enveloped viral particles have suggested a new challenge for HEV vaccine development. It was found that more than 90% of HEV particles from an HEV-infected individual’s serum were quasi-enveloped and unable to be neutralized by antibodies against the HEV capsid protein (the major target of current licensed HEV vaccine) (Takahashi et al., 2010). Thus, in foodborne hepatitis E cases or other transmission routes, it is possible that HEV particles causing infection are quasi-enveloped particles. Therefore, it is still uncertain that vaccine-induced antibodies are capable of neutralizing such quasi-enveloped HEV particles *in vivo* to subsequently confer protection in vaccine recipients under such circumstances.

## Determinants of HEV Host Tropism

Since zoonotic HEV strains have been isolated from swine, HEV and HEV-like virus have been constantly detected in a variety of mammalian hosts. Subsequently, interspecies transmission was experimentally conducted to test all HEV isolates from mammalian hosts for zoonotic characteristics or host restricted. Details regarding mammalian HEV genotypes, natural hosts, zoonotic infectivity and experimental cross-species transmission to other animals, and pathogenesis in different hosts are summarized in **Table 1**. Based on these data, human-restricted HEV genotypes (genotypes 1 and 2), zoonotic HEV genotypes (genotypes 3, 4, and 7), and animal-restricted HEV genotypes (*Orthohepevirus C*) have been observed. Therefore, it appears that both host factors and viral determinants are involved in HEV host tropism, zoonotic infection, and clinical outcomes of HEV infection.

**Table 1:** Mammalian HEV genotypes, natural hosts, zoonotic infection and cross-species transmission to other animal and pathogenesis.

Genus	Species	Genotype	Natural hosts	Infection to humans	Clinical sign of hepatitis in human	Chronicity in immunocompromised person	Experimental animal model	Pathogenesis in animal model
*Orthohepevirus*	*Orthohepevirus A*	1	Human	Yes	Acute	No	Non-human primate Rabbit(^∗^)	Clinical signs are similar to human in Non-human Primate, Only seroconversion detected in rabbit,
		2	Human	Yes	Acute	NO	Non-human primate	Clinical sign is similar to human in non-human primate
		3	Human, pig, rabbit, deer, mongoose, wild boar	Yes	Acute and chronic	Yes	Non-human primate, Swine, rabbit,	Clinically normal in inoculated animal, except viremia, viral shedding and seroconversion
		4	Human, pig, yak, wild boar	Yes	Acute and chronic	Yes	Non-human primate, swine, rabbit	No obvious clinical sign observed in non-human primate;
								Infection in swine is similar to genotype 3, Virus replication in rabbit seems to be less effective,
		5	Wild boar	Not reported	NA	NA	Not evaluated	Not evaluated
		6	Wild boar	Not reported	NA	NA	Not evaluated	Not evaluated
		7	Camel	Yes	Chronic	Yes	Not evaluated	Not evaluated
	*Orthohepevirus C*	C1	Rat	Unlikely based on non-human primate data	NA	NA	SD rat non-human primate,	Replication of virus in SD rat is not robust;
								No infection observed in non-human primate,
		C2	Ferret	Unlikely based on non-human primate data	NA	NA	Ferret; non-human primate, Wistar rat	Non-human primate and rat are not susceptible for ferret HEV;
								Sub-clinical infection, acute hepatitis, and persistent infection were observed in ferret
	*Orthohepevirus D*		Bat	Not reported	NA	NA	Not evaluated	Not evaluated


Meanwhile, bioinformatics analysis at the complete genome level for HEV genotypes 1–4 have demonstrated that divergence of zoonotic and anthropotropic genotypes occurred approximately 536 to 1344 years ago (Purdy and Khudyakov, 2010). Genotype 1 HEV appears to be a more recent genotype than zoonotic HEV genotypes, with the estimated time to the most recent common ancestor (TMRCA) of most modern lineages of HEV-1 pinpointed to ∼87–199 years ago (Purdy and Khudyakov, 2010). Meanwhile, post-divergence evolutionary rates have appeared to differ between genotypes within *Orthohepevirus A*. Specifically, genotypes 3 and 4 of HEV appear to have higher aa substitution rates over time, resulting in fewer conserved aa sites than genotype 1 HEV across the entire genome (Brayne et al., 2017). These results imply that aa substitutions may be required for adaptation of viral infectivity to new non-human hosts. This is also consistent with codon usage bias of genotypes 3 and 4 HEV, whereby codon bias within their ORFs tends to be lower than observed for genotype 1 HEV (Bouquet et al., 2012). It is postulated that these findings may reflect the requirement of zoonotic HEV genotypes to adapt to multiple hosts and therefore different cell types with distinct microenvironments and codon usage preferences (Sridhar et al., 2017). Taken together, it appears that genotype 1 HEV (and possibly genotype 2) became highly adapted to humans after divergence from zoonotic HEV and thus did not undergo further diversification. Therefore, genotype 1 HEV (and possibly genotype 2) exhibits fewer aa substitutions and higher codon use bias after losing the ability to infect animal hosts other than non-human primates.

Although one report has demonstrated that rabbits infected by genotype 1 HEV exhibited no clinical signs of hepatitis other than seroconversion (Ma et al., 2010), no other similar results have been reported. In fact, available data still favors the premise that HEV genotypes 1 and 2 are human-restricted genotypes (Pavio et al., 2015). One consistent observation is that in humans infected by both genotypes 1 and 2 HEV only develop acute hepatitis, with no chronic cases reported, while acute hepatitis cases have been reported for zoonotic HEV infection (both genotypes 3 and 4). Considering that the high anti-HEV seropositive rate among the general population results from previous zoonotic HEV infection, acute hepatitis E cases caused by zoonotic HEV should consequently be very rare. This result aligns with research showing that non-human primates experimentally infected with genotype 3 swine HEV were clinically normal (Meng et al., 1998b). Moreover, a recent study based on 123 patients with clinical hepatitis E in Scotland demonstrated no evidence for an association between variants of genotype 3 HEV and disease severity. Therefore, these results suggest that host factors rather than virus variants contribute to observed clinical phenotype during genotype 3 zoonotic HEV infection (Smith et al., 2015a). Meanwhile, other studies using non-human primates to evaluate host pathogenicity during infection by four major human-HEV genotypes have demonstrated that HEV genotypes 1 and 2 are able to cause more severe disease than genotypes 3 and 4 (Krawczynski et al., 2011). Furthermore, is notable that swine infected with a genotype 3 HEV strain isolated from an acute human hepatitis E patient did not develop clinical hepatitis and were clinically normal, which is similar to observations for genotype 3 swine HEV infection (Meng et al., 1998b). However, viral determinants that underlie the above pathogenic differences between human-restricted HEV and zoonotic HEV remain unclear. Perhaps reverse genetics-based techniques will be useful for elucidating viral factors among the various HEV genotypes that trigger pathogenic outcomes.

Among all HEV ORFs, HEV-ORF1 is the largest ORF and is considered to play an indispensable role in determining host tropism. A recent report based on gene swapping among genotypes 1 and 4 HEV infectious clones has demonstrated that HEV-ORF1 non-structural protein facilitates crossing of the species barrier (Chatterjee et al., 2016). Based on this report, chimeric virus based on a genotype 1 HEV infectious clone bearing the genotype 4 HEV-ORF1 was shown to replicate in porcine kidney cells, while the original genotype 1 HEV could not (Chatterjee et al., 2016). However, the underlying molecular mechanism that ORF1 plays to alter host cell tropism during HEV cross-species infection is not yet clear, mainly because ORF1 accounts for more than two-thirds of the entire HEV genome. Nevertheless, among all functional domains of HEV-ORF1, the hypervariable region appears to be involved in host adaptation. When the genotype 3 swine HEV and related United States human strains (US-1 and US-2) were identified and characterized, the hypervariable regions of these genotype 3 isolates were shown to be significantly larger in size than those of genotypes 1, 2, and 4 (Meng et al., 1998b). Moreover, identification of host-viral recombination events in the hypervariable region also provided further evidence for involvement of this region in cross-species infection. Notably, the novel insertion of a human ribosome protein S17 sequence into the hypervariable region of genotype 3 HEV enhanced the ability of chimeric virus to infect cells from several species (Feagins et al., 2011; Kenney and Meng, 2015b). Meanwhile, the viral RdRp-based replication complex was tested for its role in host tropism as well. In a reporter gene-based HEV replicon system comparing the replication efficiency of genotype 1 HEV in swine cells, macaque kidney and human liver cells, it appeared that translation of the ORF2 capsid gene of genotype 1 virus was severely inhibited in swine kidney cells, which led to insufficient capsid production for optimal assembly of HEV virions (Nguyen et al., 2014).

In early studies, HEV-ORF2 was initially thought unlikely to be involved in host tropism, due to its conserved status among all HEV ORFs with 85% identity within all four major HEV genotypes infect humans (Meng, 2016). Several *in vivo* studies conducted later suggested that genotype 3- or genotype 4-based chimeric viruses bearing ORF1 from genotype 1 failed to infect or establish a robust infection in swine (Feagins et al., 2011; Cordoba et al., 2012), demonstrating that HEV-ORF2 does not potentiate virus interspecies infectivity. However, this view was challenged by new observations based on infectious recombinant chimeras of genotypes 1 and 3 HEV isolates. Newer studies showed that transfer of genotype 1 capsid aa 456 to 605 (the putative virus receptor-binding region) to genotype 3 virus prevented the chimeric virus from infecting swine as expected. These results aligned with the fact that genotype 1 viruses are unable to enter swine cells (Nguyen et al., 2014) and implied that viral capsid proteins also determine host preference. However, since cellular receptors of HEV in humans or other animals are still unidentified, the link between capsid-dependent HEV host tropism and the cellular receptor participating in viral infection of various host species would be an attractive area of future HEV research.

**FIGURE 2:**
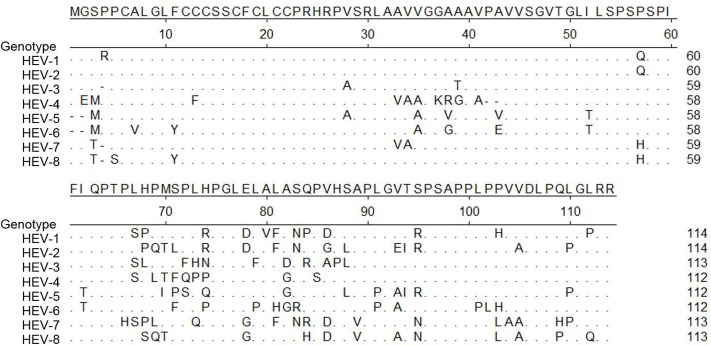
Alignment of amino acid sequence of ORF3 of eight genotypes in *Orthohepevirus A* virus. Alignment of amino acid sequence of pORF3 from all seven genotypes classified as *Orthohepevirus A* virus. Genotype 1 (GenBank accession # M73218), Genotype 2 (GenBank accession # M74506), Genotype 3 (GenBank accession # AF082843), Genotype 4 (GenBank accession # AJ272108), Genotype 5 (GenBank accession # AB573435), Genotype 6 (GenBank accession # AB602441), Genotype7 (GenBank accession # KJ496143) and Genotype8 (GenBank accession # KX387867). Those residues that are the same as consensus sequence are shown as “.”.

Besides ORF1 and 2, other studies have indicated that HEV-ORF3 product might be involved in host tropism as well. Currently, all available data suggest that HEV-ORF3 product is a class I viroporin that functions as an ion channel essential for viral particle release during HEV infection (Ding et al., 2017). The PSAP motif located within aa 95–98 is required for HEV virion release and is highly conserved among all seven genotypes of *Orthohepevirus A* (**Figure 2**). Meanwhile, our previous research indicated the first 25 aa of genotype 1 HEV are essential for HEV-ORF3’s association with microtubule, which is also involved in virus release (Kannan et al., 2009). Alignment of the ORF3 aa sequence among all eight genotypes demonstrated the first 25 aa of ORF3 are more conserved than the remaining regions (**Figure 2**). Taken together, these conserved aa sequences support a conserved role of virion release promoted by HEV-ORF3 irrespective of HEV genotype. However, there is less homology elsewhere in the protein, especially in the HEV-ORF3 C-terminal half (aa 62 to aa 114) (**Figure 2**), which appears to be important for adaptation to various hosts. Moreover, the different genomic locations of HEV-ORF3 among the species of *Orthohepevirus* (either partially overlapping ORF2 or within ORF2) (Woo et al., 2014), may suggest a genotype-specific evolution pattern influencing genotype-specific function for HEV-ORF3 to shape host tropism (**Figure 1B**). This speculation is also consistent with our previous observation of genotype-specific enhancement of IFN induction by the HEV-ORF3 product (Nan et al., 2014). How a genotype-specific function of ORF3 product affects HEV host tropism begs further investigation. Furthermore, in addition to the three well-known ORFs, the role played by the newly identified ORF4 in host tropism has not been studied but is of interest, since this protein is only conserved in genotype 1 HEV.

In addition to viral determinants, host factors, especially host immune status, may contribute to cross-species transmission of zoonotic HEV and HEV host tropisms. This is supported by establishment of HEV chronicity is animal models by administration of immunosuppressive drugs to animals or infection of another virus impairing immune system (Salines et al., 2015; Cao et al., 2017; Gardinali et al., 2017). Conversely, infection of organ transplantation receipt by camel HEV raises an interesting question regarding whether host immune status affects cross-species transmission of HEV (Lee et al., 2016), which is also consisted with observation that genotype 3 HEV could establish chronicity in a human liver chimeric mouse model (uPA^+/+^Nod-SCID-IL2Rγ^-/-^)(van de Garde et al., 2016). Therefore, although a species barrier shapes the HEV host tropism, if the immune system compromised in one species (e.g., rat), reduced immunity might confer host susceptibility to another species specific-HEV, such as ferret HEV.

Finally, age might be another factor related to the severity or virulence of acute hepatitis caused by zoonotic HEV isolates both for HEV genotypes 3 and 4. In animal studies conducted to evaluate pathogenicity of swine HEV, the animals used are generally young pigs. However, in one Japanese study evaluating zoonotic HEV pathogenesis in humans, among all 37 acute hepatitis E cases caused by HEV genotypes 3 and 4, only two cases were identified in individuals younger than 30 years of age (Ohnishi et al., 2006). In another Japanese study comparing HEV sequences of sporadic acute hepatitis E patients and viruses isolated from pig livers sold in markets, none of 17 sporadic cases was younger than 40 years of age (Okano et al., 2014). Similar observations were also reported for sporadic acute hepatitis E cases caused by zoonotic HEV worldwide (Festa et al., 2014; Inagaki et al., 2015). However, while elderly people tend to develop acute hepatitis E after zoonotic HEV infection, the underlying mechanism is still unclear. Indeed, the explanation appears not to be linked with weaker immunity upon aging, since zoonotic HEV infection, especially by genotype 3 HEV, tends to become chronic in immunocompromised individuals. Therefore, the linkage of age and severity of zoonotic HEV infection in humans needs further study.

## Conclusion and Future Perspectives

The panorama of HEV research has changed drastically with the latest findings in the field. Current research focuses on understanding virus biology, characterizing the host immune response during infection, and developing strategies for prevention of HEV as a zoonotic disease. More than two decades have passed since the report of the first complete genome sequence of the HEV prototype strain. Since then, our understanding of HEV zoonosis has remained limited. While diverse zoonotic HEV isolates exist in HEV genotypes 3 and 4, certain HEV isolates, such as genotype C1 and C2 of *Orthohepevirus C*, are only able to infect rats and ferrets, respectively. These results suggest an existence of host tropism and species barriers among various HEV isolates. However, except for the potential involvement of HEV ORF1 and the hypervariable region within HEV-ORF1, few studies have focused on these issues and information is still lacking to explain viral determinants and host factors involved in HEV host tropism and species barriers to interspecies HEV transmission.

Besides host tropism, HEV pathogenesis appears to involve a scenario more complex than predicted after discovery of HEV as a zoonotic pathogen. Different pathogenesis in humans or in experimental animal models induced by anthropotropic HEV (genotypes 1 and 2 which only cause acute hepatitis) or zoonotic HEV (genotypes 3 and 4, cause both acute and chronic hepatitis in humans and subclinical infections in animals) was recognized years ago. However, viral determinants influencing pathogenicity among anthropotropic and zoonotic HEV are still unidentified. Meanwhile, our understanding of chronic HEV infection in immunocompromised patients, as well as the occurrence of HEV-related ALF in both the general population and in pregnant women, requires additional research.

Although capsid proteins among all four major HEV genotypes share over 85% similarity, development of subunit vaccines based on recombinant HEV capsids was not achieved until 2012. In that year, the HEV239 vaccine was approved in China after being the only such vaccine to pass phase III trials in humans. Unfortunately, this vaccine, which is based on truncated capsid protein of genotype 1 HEV, is still unavailable to most of the world. Such vaccines are urgently needed in highly endemic HEV areas in spite of the fact that there is a high prevalence rate of HEV worldwide. Regardless, since the HEV239 vaccine is solely based on genotype 1 HEV, its efficiency for protecting against zoonotic HEV isolates is still unknown. Furthermore, recent discoveries about antigenic variation among HEV genotypes and quasi-enveloped viral particles hidden from neutralizing antibody suggest new challenges toward achieving high protective efficacy using any vaccine. Ultimately, development of a vaccine with efficacy against multiple HEV genotypes is still a challenging but a worthwhile goal.

## Author Contributions

YN prepared the main body of this manuscript. CW prepared all the figures and the table. QZ revised the manuscript. E-MZ designed this manuscript and supervised manuscript writing. All the authors contributed to the preparation of the final version of the manuscript and approved it for publication.

## Conflict of Interest Statement

The authors declare that the research was conducted in the absence of any commercial or financial relationships that could be construed as a potential conflict of interest.
